# The cholesterol-dependent cytolysin promotes *Streptococcus* systemic spread and induces arachidonic acid accumulation-mediated lethality in a murine intraperitoneal infection model

**DOI:** 10.1128/iai.00164-26

**Published:** 2026-06-22

**Authors:** Linya Xia, Xingyu Tian, Chen Yuan, Zhaoxuan Zhu, Fei Pan, Hongjie Fan, Karthik Hullahalli, Matthew K. Waldor, Zhe Ma

**Affiliations:** 1 Ministry of Agriculture Key Laboratory of Animal Bacteriology, the International Joint Laboratory of Animal Health and Food Safety, and College of Veterinary Medicine, Nanjing Agricultural University 70578, Nanjing, Jiangsu, China; 2 Jiangsu Co-innovation Center for Prevention and Control of Important Animal Infectious Diseases and Zoonoses 484907 , Yangzhou, Jiangsu, China; 3 Division of Infectious Diseases, Howard Hughes Medical Institute, Brigham and Women’s Hospital, Harvard Medical School, Boston, Massachusetts, USA; 4 Department of Microbiology, Harvard Medical School, Boston, Massachusetts, USA; 5 Department of Microbiology and Immunology, Stritch School of Medicine, Loyola University Chicago 12248, Maywood, Illinois, USA; University of Illinois Chicago, Chicago, Illinois, USA

**Keywords:** suilysin, bottleneck, acute death, arachidonic acid, *Streptococcus suis* serotype 2

## Abstract

Streptococcal peritonitis is associated with a high mortality risk and sometimes accompanied by streptococcal toxic shock-like syndrome (STSLS). Here, we used a murine intraperitoneal model of *Streptococcus suis* serotype 2 (SS2) infection and applied bacterial lineage tracing to investigate the pathogen’s population dynamics during streptococcal intraperitoneal infection. We found that the cholesterol-dependent cytolysin suilysin (SLY) was essential for acute lethality and facilitated bacterial systemic spread by mitigating infection bottlenecks and enabling vascular leakage in the peritoneal cavity. SLY induced marked accumulation of arachidonic acid (AA) in the peritoneal cavity. Although inhibition of AA synthesis alone did not prevent mortality, it widened the therapeutic window of antibiotic treatment, enhancing their efficacy and capacity to reduce lethality. These findings identify SLY as a critical virulence determinant linking inflammation-associated metabolic alterations, disruption of vascular integrity, and mortality and highlight AA metabolism as a promising adjunctive therapeutic target for acute SS2 intraperitoneal infection.

## INTRODUCTION

*Streptococcus suis* serotype 2 (SS2) is an opportunistic zoonotic pathogen. SS2 infections are widespread, with notably high morbidity and mortality rates reported in Asian and European countries (1–3). Although SS2 infections are commonly linked to respiratory or wound exposure, the gastrointestinal tract is also a key entry route and has been implicated in spontaneous bacterial peritonitis caused by SS2 due to pathogen translocation in patients with cirrhosis, especially in regions like Thailand and Vietnam where raw pork is consumed (4). Streptococcal peritonitis carries a high risk of mortality and is sometimes accompanied by streptococcal toxic shock-like syndrome (STSLS) (5). This syndrome is characterized by high bacterial loads, cytokine storm, with high levels of tumor necrosis factor-alpha (TNF-α), interleukin (IL)-1β, IL-6, IL-8, IL-12, and interferon-γ (IFN-γ) (6), multiple organ dysfunction, and often leads to acute host mortality (7–9). The fatality of SS2 infection can reach 12.8% (10, 11) and is generally associated with STSLS (12). Previous studies have focused on cytokine storm, but these mediators are rapidly fluctuating (13). Here, we assessed systemic inflammation by measuring C-reactive protein (CRP), a stable acute-phase protein that reflects systemic inflammatory burden (14, 15).

Suilysin (SLY), a cholesterol-dependent cytolysin (CDC) that lyses mammalian cells by forming pores in their membranes, is a key SS2 virulence factor (16). Recent studies have reported that SLY can lead to high levels of NLRP3 activation and ultimately result in STSLS in the host (6). Moreover, high SLY expression levels have been linked to cytokine storm and STSLS (17). Although the pathogenic mechanisms of SLY have received considerable attention (18–20), its role in SS2 peritonitis and relevance to death associated with intraperitoneal SS2 infection have not been reported.

Here, we used barcoded SS2 to quantify its population dynamics after intraperitoneal infection. We discovered that SLY promotes vascular leakage and influences SS2 infection dynamics. Furthermore, SLY was identified as a major driver of arachidonic acid (AA) accumulation, and AA was shown to contribute to lethality in this model. These findings provide mechanistic insight into SLY-mediated pathogenesis and suggest potential avenues for therapeutic intervention in SS2 intraperitoneal infection.

## RESULTS

### Rapid systemic dissemination and acute lethality in SS2 intraperitoneal infection

**Fig 1:**
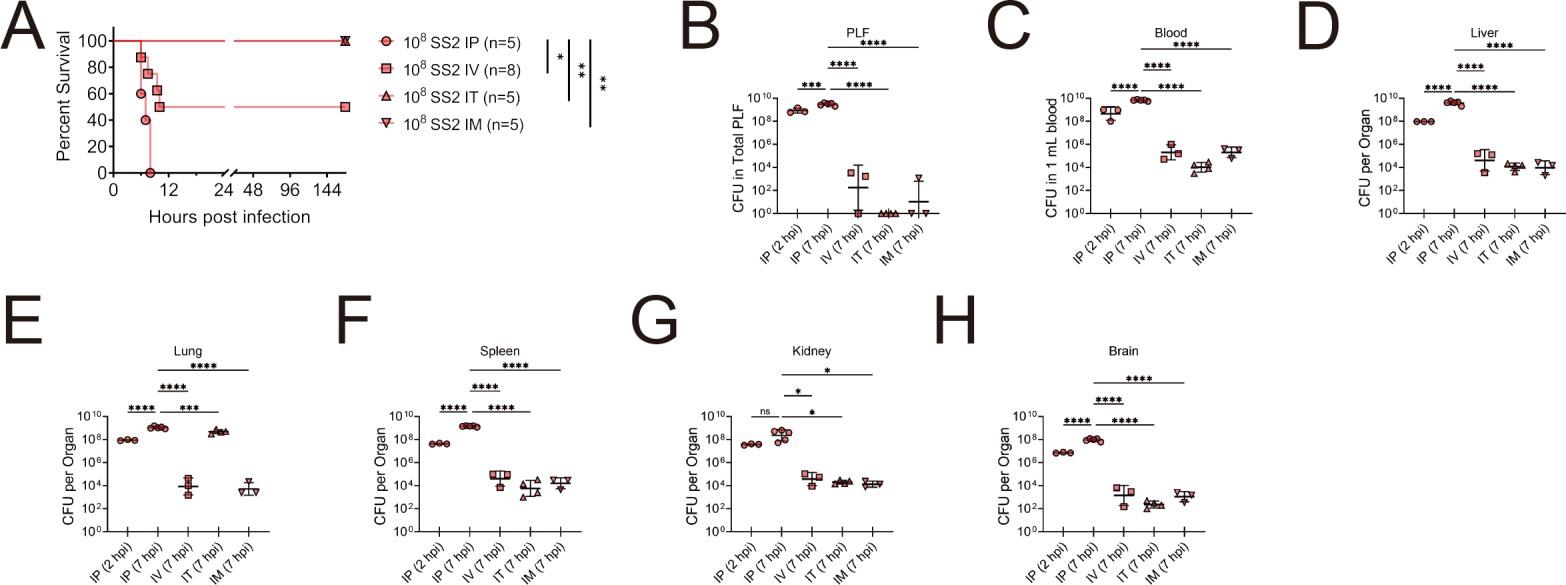
Virulence of SS2 via different infection routes in mice. (**A**) Survival curves of mice challenged with 1 × 10^8^ CFU of SS2 via different routes (intraperitoneal, IP; intravenous, IV; intratracheal, IT; or intramuscular, IM). Survival curves were analyzed using the Log-rank (Mantel-Cox) test for (**A**): **P < 0.01; *P < 0.05. (**B–H**) Bacterial CFUs in the PLF, blood, and other organs from mice challenged with 1 × 10^8^ CFU of SS2 at 2 hpi via IP (*n* = 3) or at 7 hpi via IP (*n* = 5), IV (*n* = 3), IT (*n* = 4), and IM (*n* = 3). Bars indicate geometric means with geometric SD. Statistical significance was calculated using one-way ANOVA by Tukey test against IP (7 hpi) group. The ns indicates no significant difference, ****P < 0.0001; ***P < 0.001; *P < 0.05.

SS2 can infect hosts through multiple routes, and different infection routes may lead to distinct disease manifestations (21). To determine which route of SS2 infection causes more severe disease in mice, we compared the severity of SS2 infection following different routes of administration. In these experiments, 10^8^ CFU SS2 was inoculated via intraperitoneal (IP), intravenous (IV), intratracheal (IT), or intramuscular (IM) routes. Among these, IP challenge led to the most rapid and severe disease progression, with 100% mortality observed within 8 hours post-infection (hpi). In contrast, all mice challenged via the IT and IM routes survived during the 7-day monitoring period. IV challenge resulted in intermediate lethality, with 50% of mice succumbing within 12 hpi, while the remaining animals survived (Fig. 1A). We collected peritoneal lavage fluid (PLF) to assess the bacterial burden in the peritoneal cavity following IP challenge. Bacterial counts in the peritoneal cavity reached ~10^9^ CFU by 7 hpi in moribund mice, suggesting further pathogen replication in this compartment following IP inoculation (Fig. 1B). Moreover, in mice challenged via the IP route, bacterial burdens in the blood and major organs, including the liver, lung, spleen, kidney, and brain, were already elevated at 2 hpi and remained substantially higher than those observed in the IV, IT, or IM groups at 7 hpi with the exception of lung samples from intratracheally infected mice (Fig. 1C through H). These results indicate that, compared to other routes, IP infection with SS2 results in peritoneal cavity proliferation and rapid pathogen dissemination and further triggers acute host mortality. The ability of SS2 to translocate from peritoneal cavity to blood following IP challenge likely promotes early systemic dissemination, leading to elevated bacterial burdens in multiple organs.

### Suilysin widens the bottleneck to SS2 systemic spread and increases lethality

**Fig 2:**
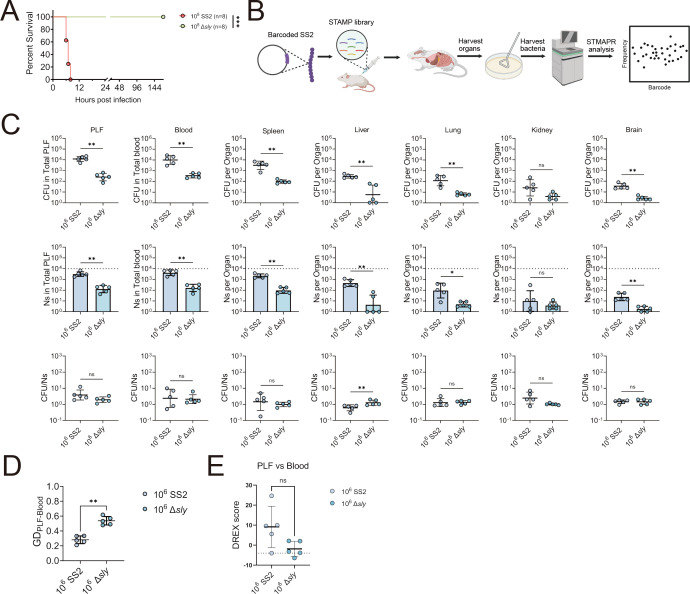
Evaluation of the influence of SLY in SS2 virulence and dissemination following IP infection. (**A**) Survival curves of mice challenged with 1 × 10^8^ CFU of SS2 or Δ*sly*. Survival animals were monitored for 7 days. The Log-rank (Mantel-Cox) test was used for statistical analysis, ***P < 0.001. (**B**) The schematic of the experimental protocol with SS2 barcoded library, each colored bacterium has a unique barcode. Mice were intraperitoneally inoculated with a genetically barcoded SS2 library. After cardiac perfusion, organs were collected, homogenized, and plated to determine CFU counts. Bacterial colonies were subsequently harvested for barcode frequency analysis (https://www.biorender.com/). (**C**) Bacterial burden (Top), the Ns (Middle), and CFU/Ns (a gage of expansion, Bottom) in PLF, blood, and organs of mice were measured after challenging with 1 × 10^6^ CFU of SS2-STAMP library or Δ*sly*-STAMP library at 2 hpi (*n* = 5). Bars indicate geometric means with geometric SD. Dotted line at 10^4^ represents the resolution limit of the standard curve of libraries. Statistical significance was calculated using nonparametric *T* test by Mann-Whitney *U* test. ns, not significant; **P < 0.01; *P < 0.05. (**D**) Genetic distance (GD) values between PLF and blood at 2 hpi with 1 × 10^6^ CFU of WT-STAMP library or Δ*sly*-STAMP library at 2 hpi (*n* = 5). Symbols are individual samples, and bars indicate means with SD. Statistical significance was calculated using nonparametric *T* test by Mann-Whitney *U* test, **P < 0.01. (**E**) DREX scores for the PLF compared to the blood. The DREX score is a metric designed to prevent misinterpretation of bacterial dissemination by accounting for the influence of high founding population sizes. Values less than −4 signify that biologically obtained GD values are less than GD values obtained by random sampling, which is not observed in these studies. Symbols are individual samples, and bars indicate means with SD. Statistical significance was calculated using nonparametric *T* test by Mann-Whitney *U* test. ns, not significant.

Since SLY is critical to SS2 pathogenicity, we compared the lethality of Δ*sly* (an SS2 *sly* gene deletion mutant) to WT SS2 following intraperitoneal administration of 10^8^ CFU. In stark contrast to the WT, the mutant did not cause death (Fig. 2A), suggesting that SLY is a critical factor for SS2 lethality following IP infection. The difference in lethality may suggest a difference in bacterial infection dynamics. To unravel SS2 population dynamics and the role of SLY in these dynamics, we used Sequence Tag-based Analysis of Microbial Population Dynamics with Re-sampling (STAMPR). Barcodes, which consist of unique short nucleotide sequence, were integrated at an identical neutral locus in the SS2 genome to create barcoded SS2 and Δ*sly* libraries (Fig. S1a). Following IP inoculation of these libraries and performing deep sequencing and STAMPR analysis (Fig. 2B) (22), we quantified the host bottlenecks during infection, which represent immune and physical barriers that restrict pathogen establishment and spread (23–25). The pathogen cells that are not eliminated by the bottleneck and instead give rise to the population at the site of infection are referred to as the founding population (FP).

The WT-STAMP and Δ*sly*-STAMP libraries exhibited similar *in vitro* growth kinetics comparable to the WT strain (Fig. S1b and c and S2a and b), and barcode stability was maintained in the absence of kanamycin (Fig. S1f and c). IP injection of 10⁸ CFU of the Δ*sly*-STAMP library did not cause mortality in mice similar to the parental Δ*sly* strain (Fig. S2d). By plating serial dilutions of the cultured libraries and comparing the CFU to the calculated FP sizes, we established that both the WT-STAMP and Δ*sly*-STAMP libraries can accurately estimate bottleneck sizes up to at least 10^4^ cells (Fig. S1d and e and S2e and f). In initial experiments, when we IP inoculated 10^8^ CFU of WT-STAMP or Δ*sly*-STAMP libraries for bottleneck analysis, we found that Ns (a metric that quantifies the FP) measurements were outside the range required for accurate assessments of FP (Fig. S3). In subsequent experiments, we reduced the challenge dose to 10^6^ CFU, enabling accurate estimate of the bottlenecks to SS2 and Δ*sly*. At 2 hpi with this lower challenge dose, we measured CFU and Ns for both strains in PLF, blood, spleen, liver, lung, kidney, and brain. To obtain the total circulating bacterial load and prevent coagulation, we collected blood via terminal cardiac perfusion and plated the entire volume for CFU enumeration, which also minimized potential confounding contributions of blood-borne bacteria when quantifying CFU in organ homogenates. Even at this early time point, CFU were recoverable from all tissue samples, but there was marked variation between organs. The highest numbers of SS2 CFU were found in the PLF and blood and were similar (~10^4^ in WT and ~10^2^ in Δ*sly*). Ns values in these samples were also similar (10^3^ to 10^4^ in WT and ~10^2^ in Δ*sly*) and the ratio of CFU/Ns, a measure of replication was less than 10 (Fig. 2C). These observations suggest that following IP inoculation, SS2 rapidly transits from the peritoneum to the blood stream and that there is relatively little replication apparent at this early time point. Therefore, the differences in bacterial burden between SS2 and Δ*sly* at 2 hpi can be interpreted as a failure of Δ*sly* to translocate from the peritoneum to the blood, rather than by heightened capacity of WT SS2 to replicate.

Bacterial loads and FP sizes in the PLF and blood observed 2 h following IP inoculation of the Δ*sly* mutant were 10- to 100-fold lower than observed with the WT strain. Thus, in the absence of SLY, the Δ*sly* population encountered a more stringent infection bottleneck than WT SS2 in these compartments where the infection initiates (Fig. 2C). CFU and Ns values were similarly reduced by 10–100 in most organs as well, likely reflecting the reductions observed in blood. In addition to quantifying FP sizes, we also compared barcodes between blood and PLF using a GD metric, which effectively quantifies dissemination of bacteria. Low GD values (close to zero) indicate high levels of barcode similarity between tissues and are suggestive of heightened dissemination. In the absence of SLY, GD values were elevated, indicating that the barcodes between blood and PLF were consistently less similar (Fig. 2D). However, increased GD values were expected based on the decrease in FP sizes, as quantified by a simulation-based metric known as the DREX score (distance from randomly sampled expectations) (Fig. 2E). Our data are consistent with a model where SLY enables heightened dissemination of SS2 across the primary barrier that prevents bacterial translocation from the peritoneal cavity to the blood.

Together these experiments with the barcoded SS2 and the Δ*sly* populations revealed that although SS2 spreads widely throughout the body, there are notable differences in the bottlenecks in different tissues; moreover, SLY has potent capacity to relax the peritoneal and blood bottlenecks.

### SLY promotes peritoneal vascular leakage during SS2 intraperitoneal infection

**Fig 3:**
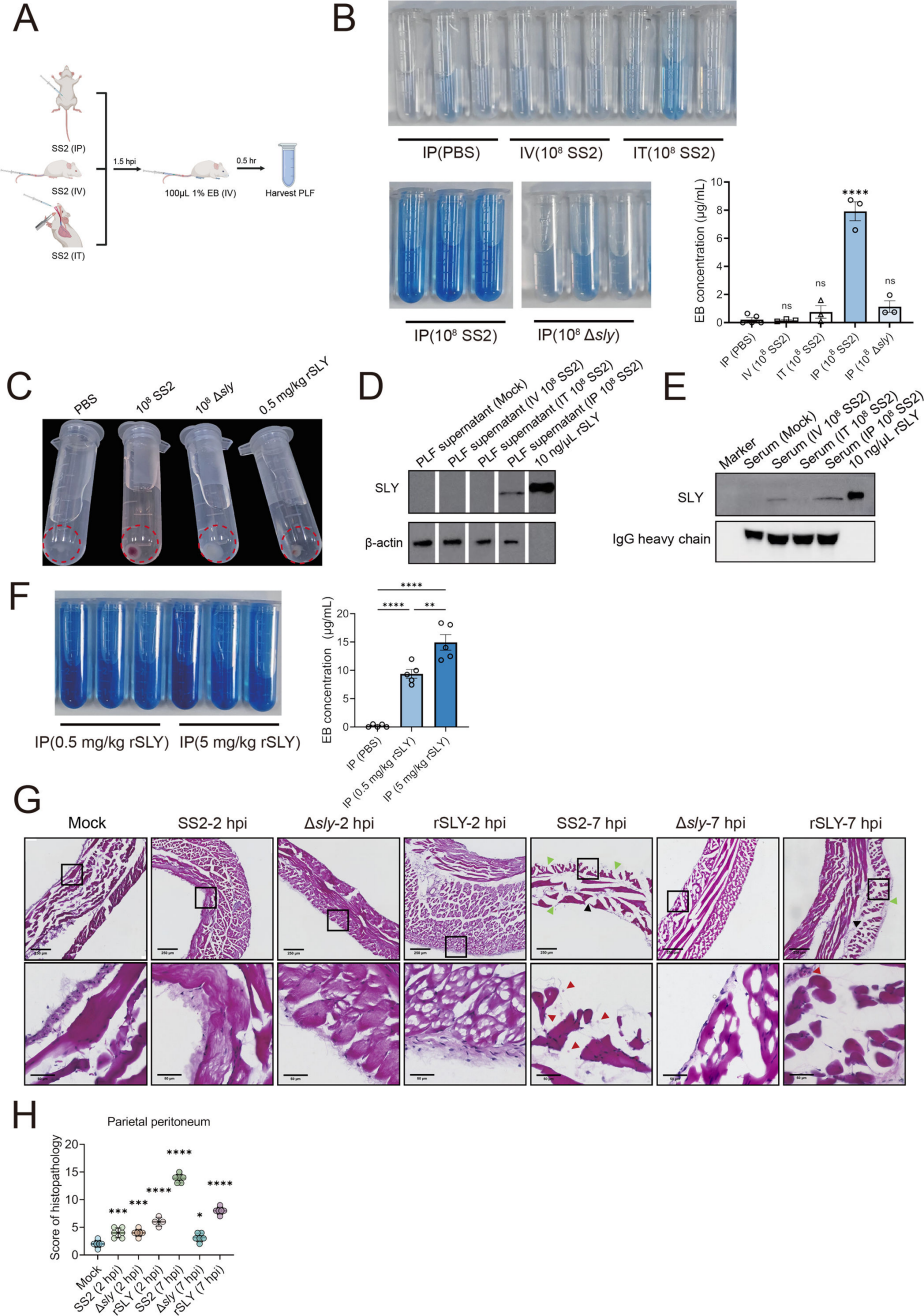
SLY accumulates in the peritoneal cavity and induces vascular leakage during SS2 intraperitoneal infection. (**A**) Schematic representation of the experimental protocol for assessing vascular permeability using Evans Blue (EB) dye. Mice were infected with bacteria via different routes. EB dye was injected intravenously via the tail vein at 1.5 hpi. Thirty minutes later, 4 mL of PBS was used to lavage the peritoneal cavity, and the recovered PLF was collected for EB quantification (created with BioRender.com). (**B**) PLF samples were collected from mice infected with SS2 or Δ*sly* through different routes, with three biological replicates per group. Cells were removed by centrifugation, and the absorbance of the supernatant at 610 nm was measured to determine EB dye concentration. Each symbol represents an individual sample. Data are presented as mean ± SEM, and statistical significance was determined by one-way ANOVA with Tukey’s multiple-comparison test against the PBS group. ns, not significant; ****P < 0.0001. (**C**) Mice were intraperitoneally injected with PBS, 1 × 10^8^ CFU SS2, 1 × 10^8^ CFU Δ*sly*, or 0.5 mg/kg rSLY. PLF samples were collected at 2 hpi and centrifuged at 1,500 *g* for 10 min. (**D and E**) SLY was detected by Western blot in the PLF supernatant (**D**) and serum (**E**) from mice infected with SS2 via different routes at 7 hpi. rSLY served as a positive control, and PLF from uninfected mice served as a mock control. (**F**) PLF samples were collected from mice administered 0.5 mg/kg or 5 mg/kg rSLY intraperitoneally, with three biological replicates per group. Cells were removed by centrifugation, and the absorbance of the supernatant at 610 nm was measured to determine EB dye concentration. Each symbol represents an individual sample. Data are presented as mean ± SEM, and statistical significance was determined by one-way ANOVA with Tukey’s multiple-comparison test. ****P < 0.0001; **P < 0.01. (**G**) Representative hematoxylin and eosin (H&E) stained sections of parietal peritoneum from mice treated with 1 × 10^8^ CFU SS2, 1 × 10^8^ CFU Δ*sly*, or 0.5 mg/kg rSLY at 2 or 7 hpi. PBS was used in the mock group. Enlarged regions are indicated by black squares. Green arrows denote disrupted mesothelial cells, black arrows indicate degenerated connective tissue, and red arrows indicate extravasated erythrocytes. (**H**) Histopathological scoring based on H&E staining. Data are presented as mean ± SD, and statistical significance was determined by one-way ANOVA with Dunnet’s multiple-comparison test against the mock group. ****P < 0.0001; ***P < 0.001; *P < 0.05.

Evans Blue (EB) dye is widely used as a tracer to assess vascular permeability *in vivo* (26). This dye was injected via the tail vein at 1.5 hpi with 10^8^ CFU SS2 inoculated through different routes, and PLF was collected 0.5 h later (Fig. 3A). Notably, the amount of EB in the PLF of IP challenged mice was markedly higher than found in IV or IT challenged animals, suggesting that SS2 installation into the peritoneal cavity induces pronounced vascular leakage into the peritoneum as early as 2 hpi (Fig. 3B). In stark contrast, IP inoculation of 1 × 10^8^ CFU Δ*sly* led to significantly lower EB amounts in the PLF compared to SS2 WT infection (Fig. 3B), strongly implicating SLY in promoting vascular leakage into the peritoneum. Together these observations suggest that SLY damages the vascular integrity the peritoneal cavity, which, in turn, facilitates leakage of fluid into the peritoneum as well as bacterial translocation from the peritoneal cavity.

The presence of red blood cells in the PLF can serve as another indicator of vascular leakage. Red cell pellets were observed in PLF from mice challenged intraperitoneally with SS2 WT or rSLY, recombinant SLY protein (Fig. S4a and b), but not in those infected with the Δ*sly* mutant (Fig. 3C). Western blot analysis of samples from WT-infected mice revealed that SLY was detectable in both the PLF supernatant (removing cells by centrifugation) and serum following IP challenge at 7 hpi (Fig. 3D and E). In contrast, although SLY was detected in the serum of IV-infected mice, it was undetectable in their PLF. No SLY was detected in either the serum or PLF of mice infected via the IT route (Fig. 3D and E). Administration of as low as 0.5 mg/kg dose rSLY into peritoneal cavity resulted in EB extravasation into the PLF (Fig. 3F). We performed histopathological analyses of the parietal peritoneum (Fig. 3G and H). Intraperitoneal administration of either rSLY or SS2 led to marked vascular injury, characterized by erythrocyte extravasation and inflammatory infiltration; in contrast, ip inoculation of Δ*sly* led to far less damage (Fig. 3G and H). Thus, SLY causes marked damage to the peritoneum, providing a likely morphological basis for the observed EB leakage. Collectively, these findings suggest that SLY helps widen the bottleneck for SS2 dissemination from the peritoneal cavity into the systemic circulation, likely, in part, through its ability to promote peritoneum vascular leakage.

### SLY induced arachidonic acid elevation may contribute to severe inflammatory responses

Since acute death caused by SS2 infection is closely associated with inflammation and cytokine storms, we assessed the inflammatory response by measuring CRP levels in hosts infected with SS2 WT or Δ*sly* intraperitoneally. Unlike rapidly fluctuating cytokines, CRP is a stable acute-phase protein that reliably reflects systemic inflammatory burden, making it a more consistent marker of overall inflammation during this critical time course (14, 15). At a challenge dose of 1 × 10^8^ CFU, the WT strain elicited a marked increase in CRP, indicative of an inflammatory response, whereas the Δ*sly* mutant did not (Fig. S5).

**Fig 4:**
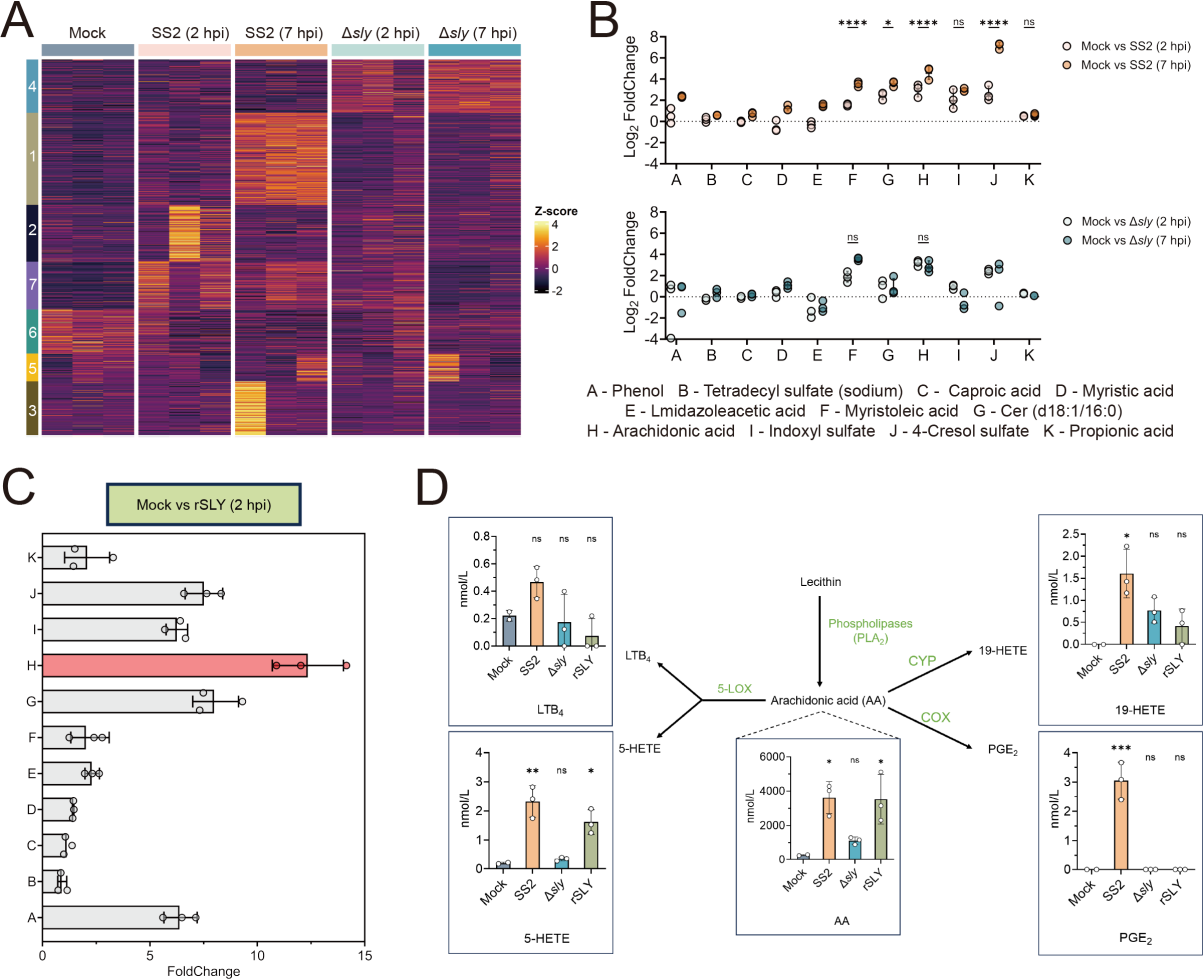
Metabolomic analysis of SLY-mediated alterations in peritoneal cavity metabolites. (**A**) Unsupervised clustering (k-medoids) of metabolites identified in the PLF of mice challenged with SS2 or Δ*sly* at 2 or 7 hpi (*n* = 3 per group). Mock samples were collected from uninfected mice (*n* = 3). Metabolites were categorized into seven clusters based on relative abundance patterns. Each row represents one metabolite, and each column represents a sample (including biological replicate). (**B**) Relative abundance fold-change of 11 proinflammatory metabolites identified in Clusters 1 (criteria: upregulated, *P* < 0.05, and known proinflammatory function). Upper panel: mock vs SS2 groups at 2 and 7 hpi. Lower panel: mock vs Δ*sly* groups at 2 and 7 hpi. Eleven proinflammatory metabolites codes are Phenol (A), Tetradecyl sulfate (sodium) (B), Caproic acid (C), Myristic acid (D), Lmidazoleacetic acid (E), Myristoleic acid (F), Cer (d18:1/16:0) (G), Arachidonic acid (H), Indoxyl sulfate (I), 4-Cresol sulfate (J), Propionic acid (K). Bars indicate geometric means with geometric SD. Statistical significance was determined using two-way ANOVA with Bonferroni’s multiple-comparison test. ns, not significant; ****P < 0.0001; *P < 0.05. (**C**) The relative abundance fold-change of the 11 proinflammatory metabolites in mice treated with 0.5 mg/kg rSLY at 2 hpi compared with the mock group. (**D**) Targeted metabolomic quantification of oxidized lipids in PLF from mice challenged intraperitoneally with 1 × 10^8^ CFU WT SS2, Δ*sly*, or with 0.5 mg/kg rSLY, at 7 hpi. 5-HETE, LTB_4_, 19-HETE, and PGE_2_ are downstream products of the three major arachidonic acid (AA) metabolic pathways associated with proinflammatory activity. Each symbol represents an individual mouse. Data are presented as mean ± SD, and statistical significance was evaluated by one-way ANOVA with Tukey’s multiple-comparison test against the mock group. ns, not significant; ***P < 0.001; **P < 0.01; *P < 0.05.

Although CRP elevation confirmed the presence of a systemic inflammatory response, the mechanisms driving rapid lethality within 8 h remained incompletely understood. Previous studies have focused on cytokine storm (6, 17). In other models, these inflammatory mediators are often driven by upstream metabolites (27). To comprehensively profile the *sly*-dependent metabolites of the peritoneal microenvironment after infection, PLF samples were collected from three groups, including uninfected, WT-infected, and Δ*sly*-infected mice at 2 and 7 hpi, respectively, followed by untargeted metabolomic analysis. A total of 850 metabolites were identified and classified into 12 major categories according to first-level annotation (Fig. S6a and b). Comparison of the infected group and the mock group further allowed classification of all metabolites into seven clusters according to abundance patterns, using unsupervised k-medoids clustering (Fig. 4A). Cluster 1 metabolites showed higher abundance at 7 hpi in WT-infected mice, and we identified 11 metabolites with confirmed proinflammatory functions in this group (28–38). Notably, in WT-infected mice, four metabolites, Myristoleic acid, Cer (d18:1/16:0), Arachidonic acid (AA), and 4-Cresol sulfate, showed a markedly higher log_2_ Fc at 7 hpi vs mock than at 2 hpi vs mock group. Although several of these metabolites increased in the Δ*sly*-infected mice compared to the mock (log_2_ Fc > 1), their log_2_ Fc at 2 hpi vs mock group did not differ significantly from their log_2_ Fc values at 7 hpi vs mock. These findings suggest that the accumulation of these four metabolites in PLF represents a SLY-dependent metabolic response during SS2 intraperitoneal infection (Fig. 4B).

We further performed untargeted metabolomic analyses of PLF samples from mice inoculated with rSLY at 2 hpi. Compared with the mock group, AA showed the highest fold change among the 11 inflammation-associated metabolites, increasing by ~12-fold (Fig. 4C), further supporting the role of SLY in driving AA accumulation. Untargeted metabolomic analysis also revealed a marked upregulation of lecithin (C00157), the primary precursor of AA (C00219), in the WT- and rSLY-treated groups, together with several other lipid or lipid-like metabolites associated with lecithin biosynthesis (Fig. S6c and d). AA, as a central precursor for a series of steroid hormones, is converted into various pro-inflammatory downstream products that mediate the inflammatory response through three main enzymatic pathways: cyclooxygenase (COX), lipoxygenase (LOX), and cytochrome P450 (CYP) (39). We measured AA and its downstream inflammation-associated metabolites in the PLF of mice infected with WT or Δ*sly* or treated with rSLY. SS2 infection elevated AA and its four downstream metabolites, whereas these increases were markedly attenuated in Δ*sly*-infected mice. However, with the exception of 5-HETE generated through the 5-LOX pathway, rSLY treatment did not reproduce the elevation of the other three metabolites, suggesting that additional bacterial factors may modulate AA metabolism toward the generation of inflammation-associated mediators (Fig. 4D).

### Inhibition of AA production may enhance antibiotic efficacy in acute lethal SS2 intraperitoneal infection

**Fig 5:**
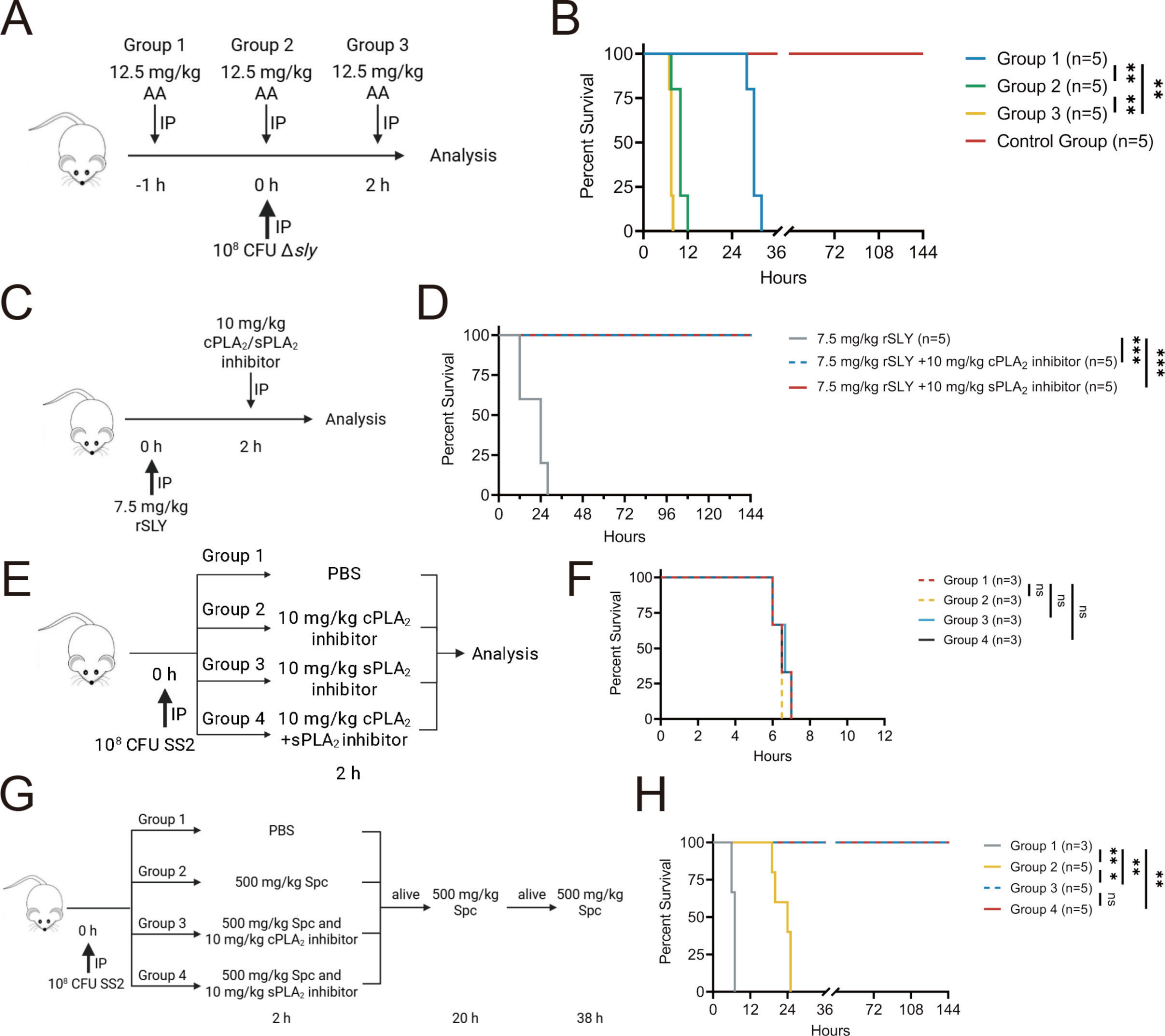
Survival analysis of mice treated with PLA_2_ inhibitors and antibiotics during SS2 infection. (**A and B**) Experimental schematic (**A**) and survival curves (**B**) showing the effects of exogenous AA administration in Δ*sly*-infected mice. Mice were infected intraperitoneally with 1 × 10^8^ CFU Δ*sly*. AA (12.5 mg/kg) was administered intraperitoneally 1 h before, at the time of, or 2 h after infection. The control group received AA only. (**C and D**) Experimental schematic (**C**) and survival curves (**D**) of mice co-administered with rSLY and PLA_2_ inhibitors. Mice were injected intraperitoneally with 7.5 mg/kg rSLY, followed by intraperitoneal administration of either a cPLA_2_ inhibitor or an sPLA_2_ inhibitor (10 mg/kg) at 2 hpi. (**E and F**) Experimental schematic (**E**) and survival curves (**F**) showing the effects of PLA_2_ inhibition during WT SS2 infection. Mice were infected intraperitoneally with 1 × 10^8^ CFU SS2, 10 mg/kg of either cPLA_2_ or sPLA_2_ inhibitor, and a combination of 10 mg/kg cPLA_2_ inhibitor and 10 mg/kg sPLA_2_ inhibitor was administered intraperitoneally at 2 hpi. (**G and H**) Experimental schematic (**G**) and survival curves (**H**) of therapeutic effects combining PLA_2_ inhibitors with antibiotics. Mice were infected intraperitoneally with 1 × 10^8^ CFU WT SS2. Treatments were initiated at 2 hpi, with one group receiving spectinomycin (Spc) alone and another receiving Spc in combination with sPLA_2_ or cPLA_2_ inhibitors. Subsequent Spc was administered at 18 h interval to surviving mice. Survival data were statistically analyzed using the log-rank (Mantel-Cox) test. ns, not significant; ***P < 0.001; **P < 0.01; *P < 0.05.

To assess the role of AA in SS2 lethality in the absence of SLY, mice were intraperitoneally administered AA either 1 h before, simultaneously with, or after Δ*sly* infection (Fig. 5A). In marked contrast to what was observed in the absence of AA treatment (Fig. 2), all animals given AA succumbed to infection (Fig. 5B); AA potentiation of SS2 lethality was more effective when AA was given at the time of or 2 h after ip Δ*sly* infection. These findings suggest that SS2 production of SLY in the peritoneum leads to the elevation of AA, which contributes to SS2 lethality.

To investigate whether inhibition of AA production could mitigate SLY-induced SS2 lethality, AA synthesis inhibitors were administered in a murine challenge model. Phospholipase A_2_ (PLA_2_) is the key rate limiting enzyme that catalyzes the hydrolysis of membrane phospholipids to generate AA. Among its isoforms, secretory PLA_2_ (sPLA_2_) and cytosolic PLA_2α_ (cPLA_2α_) are two major subtypes. The AA-generating cascade is thought to be initiated by cPLA_2α_ and maintained through sPLA_2_ activation, which may amplify AA release and inflammatory responses during infection (40, 41). Intraperitoneal administration of rSLY caused 100% mortality in mice within 36 h. However, treatment with alminoprofen (a sPLA_2_ inhibitor) or D-erythro-dihydrosphingosine (a cPLA_2_ inhibitor) reduced the mortality rate to 0%, with all mice surviving the otherwise lethal rSLY challenge (Fig. 5C and D), showing that inhibition of either enzyme effectively disrupts this pathway, preventing excessive AA accumulation and SLY-mediated lethality. To test whether AA induces pro-inflammatory cytokine expression *in vivo*, we injected mice with AA (12.5 mg/kg) or rSLY (7.5 mg/kg) and analyzed IL-1β, IL-18, and TNF-α mRNA levels in PLF cells at 7 hpi. Both rSLY and AA upregulated these cytokines compared to mock controls, confirming that AA is sufficient to trigger inflammatory gene expression (Fig. S7a through c).

To further evaluate the therapeutic potential of inhibiting AA production during SS2 intraperitoneal infection, we tested the effects of the two AA synthesis inhibitors on SS2-induced lethality. Treatment with either inhibitor alone did not prevent mortality, as WT SS2 still caused 100% death (Fig. 5E and F), indicating that monotherapy with these inhibitors is insufficient. To determine whether combined inhibition of both PLA_2_ isoforms offers additional benefit, we treated WT SS2-infected mice with cPLA_2_ and sPLA_2_ inhibitors simultaneously. Dual inhibition did not improve survival compared with either inhibitor alone (Fig. 5E and F).

We next assessed combination therapy with either inhibitor and the antibiotic spectinomycin (Spc) (42). Spc monotherapy only transiently prolonged survival, delaying 100% mortality from 8 h in the control group to 24 h even though survivors at 20 hpi received a second dose of Spc. Notably, when Spc was combined with either inhibitor, all mice survived through the second Spc treatment at 20 hpi and the third treatment at 38 hpi. By the end of the 144-h observation period, 100% of mice receiving the combination therapy survived (Fig. 5G and H), suggesting that inhibition of AA production not only effectively prolongs survival but also widens the therapeutic window of antibiotic treatment, enabling 100% of survival of an infection that was uniformly fatal without the adjunctive inhibition of AA production.

To investigate whether the protective effect of PLA_2_ inhibitors involves enhanced bacterial clearance, we quantified bacterial loads in PLF, blood, and organs at 7 hpi. Spc treatment alone significantly reduced bacterial loads in all compartments compared to untreated controls. However, the addition of either cPLA_2_ or sPLA_2_ inhibitor to Spc treatment did not further reduce bacterial loads (Fig. S7d through j). These data indicate that the survival benefit conferred by PLA_2_ inhibitors in combination with antibiotics is not attributable to enhanced bacterial clearance. Our findings support a model in which PLA_2_ inhibition mitigates AA-driven inflammatory pathology, thereby enhancing the therapeutic effect for antibiotics to control the infection.

## DISCUSSION

Epidemiological investigations have reported that among clinical cases of STSLS associated with SS2 infection, mortality rates can be 97.4% (12). While the pore-forming toxin SLY is recognized as a key factor contributing to STSLS (17), its involvement in the rapid lethality associated with intraperitoneal infection has not been clearly defined. Our results are consistent with a model in which SLY facilitates SS2 systemic spread from the peritoneal cavity by widening host bottlenecks that ordinarily restrict pathogen egress from the peritoneum. Moreover, our findings suggest that SLY opens the bottleneck by damaging the vessel lining the peritoneum, enabling the pathogen to gain access to the blood and spread throughout the body. SLY may compromise vascular integrity in the peritoneal cavity through its pore-forming activity. Notably, these observations are parallel to pneumolysin (PLY) from *Streptococcus pneumoniae*, which induces pulmonary edema by disrupting the alveolar-capillary barrier (43).

SS2 likely disseminated to all the tissues we tested via the blood stream. The CFU burden and FP sizes of the spleen were similar to the blood, whereas all the other organ samples had smaller burdens and FP sizes than the blood. The similarity of the FP in blood and the spleen may reflect the spleen function to filter blood-borne pathogens; apparently splenic innate immune functions are relatively ineffective against SS2 at this early time point since the CFU burden is similar to blood. The liver, like the spleen, functions to filter the blood; however, the lower Ns value observed in the liver vs the spleen suggests that the innate immune processes restricting the pathogen population in the liver are more potent than in spleen, potentially due to the liver’s sinusoidal-resident macrophages (Kupffer cells). In the three other organs sampled, lung, kidney, and brain, CFU and Ns values were 1–2 logs lower than in the other compartments and CFU/Ns ratios were close to 1, indicating that there has been little replication. The low bacterial founder numbers observed in the kidney and lung at 2 hpi may reflect limited capacity for pathogen filtration, possibly due to the absence of dense phagocytic networks in these two organs. In the brain, low colonization levels may instead result from the restrictive properties of the blood-brain barrier, which impede pathogens entry from the circulation.

Beyond the route-dependent differences in bacterial burdens across organs (Fig. 1B and C and Fig. 3B), organ-specific microenvironments, such as nutrient availability and immune pressure, may also shape SLY expression (44, 45). In turn, differences in organ susceptibility to SLY-mediated cytotoxicity may lead to distinct pathological outcomes (46).

The peritoneal cavity and bloodstream behave almost as a continuous compartment during the first hours of infection, making it challenging to attribute dissemination defects solely to changes in tissue-specific bottlenecks. Moreover, because Δ*sly* mutants proliferate less efficiently at the inoculation site, some downstream dissemination phenotypes may reflect altered source dynamics rather than direct effects on barrier traversal. This study is the discrepancy between the infection dose required to reproduce the acute-lethality phenotype (10^8^ CFU) and the lower dose (10^6^ CFU) required for accurate STAMPR analysis. At 10^8^ CFU, the FP exceeds the upper quantification range of Ns, making it impossible to resolve bottlenecks using barcoded libraries at this physiologically relevant dose. Consequently, the STAMPR results obtained from 10^6^ CFU infection accurately reveal the qualitative role of SLY in widening infection bottlenecks, but they may not fully reproduce the population dynamics that occur under the 10^8^ CFU condition in which acute death is observed. However, extensive barcoding studies across pathogens show that FP size scales with inoculum and, importantly, that relative differences between WT and mutants are generally preserved across doses (24). This dose-independent consistency supports the interpretation that the reduced dissemination of Δ*sly* reflects a genuine bottleneck defect even though the precise population dynamics at 10^8^ CFU cannot be resolved with STAMPR.

Previous studies have largely attributed SS2 lethality to cytokine storm, involving excessive production of IL-1β, IL-18, TNF-α, IL-17A, IFN-γ, and other proinflammatory cytokines (6, 47). However, our findings suggest that additional inflammatory mediators may play even more critical roles in driving acute death associated with SS2 intraperitoneal infection. SLY appears to serve as an upstream trigger leading to the generation of AA. Through its pore-forming activity, SLY disrupts cell membrane integrity and facilitates phospholipid breakdown (48), which likely activates cPLA_2α_. Activated cPLA_2α_ initiates the release of AA and lysophospholipids, which, in turn, induce the expression and secretion of sPLA_2_, further hydrolyzing phospholipids and releasing more AA (41, 49). AA serves as a key substrate for the synthesis of inflammatory mediators, its elevation may contribute to enhanced inflammatory responses, and it may represent one of the triggers underlying SS2 infection-associated hyperinflammation (50, 51). Our finding that exogenous AA directly upregulates IL-1β, IL-18, and TNF-α expression *in vivo* further supports a causal role for AA in driving the inflammatory response during SS2 infection. In particular, AA and several downstream inflammation-associated metabolites were elevated in SS2-infected mice but not in the Δ*sly* group, suggesting that pathogenesis of SS2 infection likely involves the generation of inflammation-related lipid mediators as well as cytokines.

In summary, here, we established a murine model of acute death induced by intraperitoneal infection with SS2. Using this model and leveraging STAMPR analysis, we discovered that SLY mitigates host bottlenecks that restrict SS2 dissemination from the peritoneal cavity to the bloodstream via a process that may be explained, at least in part, by SLY-induced vascular leakage. In addition, we found that the presence of SLY led to elevated levels of AA and its downstream inflammation-associated metabolites during infection. Although inhibition of AA production alone did not prevent mortality caused by SS2 intraperitoneal infection, it widened the therapeutic window of antibiotic treatment, enhancing antibiotic efficacy and reducing lethality. Thus, this study offers new insights for the development of new interventions for SS2 intraperitoneal infection, suggesting that therapeutic strategies should not only target the pathogen but also address the inflammatory responses driven by AA elevation.

## MATERIALS AND METHODS

### Animal experiments

Five- to 6-week-old female BALB/c mice were purchased from Qinglong Mountain Company (Nanjing, China) and maintained under specific pathogen-free conditions at the Nanjing Agricultural University Experimental Animal Facility. All animal experiments were approved by the Laboratory Animal Welfare and Ethics Committee of Nanjing Agricultural University (approval number NJAU. No. 20211112168). SS2 (1×10^8^ CFU) in PBS were injected via different routes: intraperitoneal (IP) injection (100 µL), intravenous (IV) injection (100 µL), intratracheal (IT) injection (50 µL), or intramuscular (IM) injection (100 µL). PLF was collected after two successive washes with 4 mL PBS. PLF, blood, and organs were harvested and homogenized for CFU counting on THB agar plates.

As for STAMPR samples, intraperitoneal lavage and cardiac perfusion (with 15 mL PBS containing 85 µmol/L sodium citrate) were performed following anesthesia with pentobarbital at a dose of 5 mg/kg. Then, PLF, blood, and organs were harvested and homogenized for CFU counting on THB agar plates or STAMP library collection on THB agar plates with 150 µg/mL Kan.

### Construction of bacterial strains

The deletion of *sly* in SS2 was achieved using a temperature-sensitive allelic exchange vector, pSET4s. To generate the recombinant vector, the upstream and downstream regions of the *sly* gene were amplified by PCR with the primer pairs *sly*-F1/*sly*-R1 and *sly*-F2/*sly*-R2, using SS2 genomic DNA as the template. The upstream and downstream PCR products and *Bam*H I- and *Hind* III- digested linear pSET4s were mixed and assembled with Gibson assembly. The recombinant plasmid was then electroporated into SS2 (Bio-Rad, Gene Pulser Xcell, Voltage: 2,000 V, Capacitance: 25 μF, Resistance: 200 Ω, Cuvette: 1 mm), and mutant isolation was carried out as previously described (52). The complemented strain (cΔ*sly*) was cloned from mutant strain Δ*sly* with slight modifications in the plasmid construction step. Briefly, the upstream and downstream fragments and sly gene fragment containing a silent mutation (to distinguish WT and cΔ*sly*) were amplified from the ZY05719 genome and then cloned into digested pSET4s.

### Construction of barcoded SS2 STAMP library in WT and Δ*sly* strains

To create a donor plasmid containing 20 bp random nucleotides, we amplified a Kan resistance cassette from pMar4s with primers containing 20 “N” nucleotides (53). Based on previous studies, we identified a neutral site located between ZY05719_07175 and ZY05719_07180 in the SS2 (54). To introduce barcodes at this neutral site, we amplified a 145 bp fragment that aligned to the downstream of ZY05719_07175 gene with primer 07175-F/07175-R and then cloned this fragment into pSET4s with *Bam*H I and *Eco*R I to construct plasmid pSET4s-145. The 20 bp random nucleotides barcodes were induced upstream of the Kan resistance cassette with PCR with primers Kan-F1/Kan-R1. Then, the barcode-containing amplicon was inserted downstream of 145 bp fragment in pSET4s-145 with Gibson assembly to create the pSET4s-STAMP pool. The Gibson assembly was electroporated into *E. coli* DH5α on LB agar with 50 µg/mL Spc across a total of 20 petri dishes. Ten clones were picked for PCR amplification and Sanger sequencing to confirm the insertion of a random barcode sequence. Approximately 30,000 colonies were harvested, and plasmids were extracted.

The pSET4s-STAMP plasmids were electroporated into SS2 ZY05719 WT or Δ*sly*. Samples were subsequently plated onto THB agar with Spc (50 μg/mL) and Kan (50 µg/mL) and cultured for 36 h at 28°C. Approximately 2,000 colonies were picked individually and inoculated in THB with Spc (50 μg/mL) and Kan (50 µg/mL) in 96-well plates for an overnight culture at 37°C. To ensure that all cells contained the barcode sequence, these cultures were passaged in THB with 150 μg/mL Kan at 37°C overnight three times. Ten individual colonies were picked for PCR amplification and Sanger-sequenced to confirm that each they contain unique barcodes. Finally, 919 individual colonies were collected in SS2 WT-STAMP library, and 952 individual colonies were collected in Δ*sly*-STAMP library. All libraries were frozen in PBSG at −80°C (Fig. S1a).

### Quality control of barcoded libraries

To determine whether the integrated sequences affect the growth rates of barcode strains, we selected random colonies and cultured them in THB medium with the WT SS2 as control. Cells were washed once in PBS and adjusted to OD_600_ to 1.0. Cells were then inoculated 1:100 into THB medium with or without Kan (150 μg/mL). The cultures were incubated overnight at 37°C with shaking at 180 rpm, and the OD_600_ was measured to assess the growth of the strains. Insertion of barcode sequences did not influence the growth rate of WT-STAMP and Δ*sly*-STAMP libraries compared to the WT SS2 (Fig. S1b and c and S2a and b).

To evaluate whether the integrated sequences are maintained in the absence of antibiotic selection, we cultured barcoded strains in the STAMP library under conditions lacking Kanamycin. The cultures were diluted 1:1,000 and passaged continuously for at least 12 days. Every 2 days, aliquots were separately plated onto THB agar plates, both with and without Kan. The WT-STAMP and Δ*sly*-STAMP libraries were consistently stable in the absence of antibiotic selection for at least 12 days *in vitro* (Fig. S1f and c).

### STAMP sample processing

Bacterial samples obtained from either *in vitro* cultures or organ homogenates were plated on THB agar plates containing Kan (150 μg/mL) and incubated overnight at 37°C. Bacteria were harvested from the plates using PBS and subsequently stored at −80°C. Genomic DNA templates were prepared by diluting bacterial samples in water (1:50) and boiling at 95°C for 15 min. One microliter of the sample was used as the template for PCR amplification (with primers P5/P7), and cycling conditions for PCR were the following: initial denaturation 95°C 300 s, 27 cycles of 95°C 15 s, 55°C 15 s, and 72°C 10 s, and final extension of 72°C 5 min. Reactions were carried out with a total volume of 50 μL using 2 × Rapid Taq Master Mix (Vazyme, China). The presence of amplicons was pooled, verified, and purified by agarose gel extraction (Omega, USA). Purified amplicons were quantified by Qubit dsDNA HS Assay Kit (Thermo Fisher, USA) and sequenced with NovaSeq (Illumina). FASTQ files were then processed and analyzed using the STAMPR analysis pipeline to yield measurements (Ns, GD, DREX score) (22, 55–58). Ns values represent the size of bacterial founding populations. GD values quantify the similarity of tag frequencies between organs, with higher GD indicating greater dissimilarity and lower GD indicating greater similarity. DREX values were used to assess the extent to which GD values are expected by random chance, providing a reference for evaluating the significance of biologically observed GD values.

### Construction of STAMP reference barcode list and standard curve

The DADA2 algorithm was employed to deduplicate the reads, thereby generating a preliminary list of reference barcodes. We selected 919 barcodes that best matched the number of picked clones for the WT-STAMP library. The Δ*sly*-STAMP library consisted of 952 barcodes. Both libraries were employed throughout this study. The reference barcode list was then used as reference for mapping samples in the standard curve, confirming that this list of reference barcodes provides accurate founding population estimates.

STAMP libraries consisting of 10-fold serial dilutions were plated on THB agar plates supplemented with Kan (150 μg/mL), corresponding to cell concentrations ranging from 10^0^ to 10^8^ cells. These samples were utilized to generate a standard curve and served as controls with known *in vitro* bottleneck sizes, and the plated CFU reflects the observed founding population size (Fig. S1d and e). Three replicates of sample were prepared and sequenced as described above. We constructed a standard curve which showed robust correlation between the observed founders (CFU) and the calculated founding population via barcode sequencing (Ns) up to 10^4^ founders (Fig. S1e and f).

### Assessment of vascular leakage

Vascular permeability was assessed using the Evans Blue leakage assay to evaluate the extent of vascular leakage into the peritoneal cavity. After 1.5 h of different treatments, each mouse was intravenously injected with 100 μL of 1% Evans Blue via the tail vein. Thirty minutes later, mice were euthanized, and the peritoneal cavity was washed twice with PBS. PLF samples were centrifuged at 1,500 *g* for 10 min. A total of 4 mL of PLF supernatant was collected, and photographs were taken. The Evans Blue leakage was quantified using a microplate reader at OD_610_.

### HE staining

For staining, parietal peritoneum was fixed in 10% neutral buffered formalin and processed for frozen embedding. Sections with a thickness of 10 μm were cut and stained with hematoxylin and eosin. The corresponding Hematoxylin and Eosin slide to the tested tissue block were reviewed and scored jointly by two pathologists, as outlined in Table S2.

### Western blot analysis

Immunoblot analysis of CRP was carried out using serum (59). All serum samples were diluted at a 1:10 ratio using PBS. The diluted samples were subsequently mixed with loading buffer and denatured by placing the tubes in the 95°C heating block for 10 min. For each sample, 5 μL was loaded into the SDS-PAGE gel and then transferred a PVDF membranes (10 V, 40 min). Mouse IgG antibody anti-CRP was diluted 1:2,500, and HRP-labeled Goat anti-Mouse IgG antibody (Thermo Fisher, USA) was diluted 1:5,000. Secondary antibodies were detected using an enhanced ECL detection kit (Vazyme, China). Signal monitoring and quantification were performed with a Bio-Rad Chemidoc system and analyzed using Image Lab software 3.0 (Bio-Rad).

For the detection of suilysin, rabbit anti-Suilysin (*Streptococcus Suis* serotype 2) monoclonal antibody (1:5,000 dilution) and HRP-labeled Goat anti-Rabbit IgG antibody (1:5000 dilution) were used.

### Untargeted metabolomic analysis

Five- to six-week-old female BALB/c mice were injected with PBS (100 μL), SS2 (1 × 10^8^ CFU in 100 μL), Δ*sly* (1 × 10^8^ CFU in 100 μL), or rSLY (0.5 mg/kg in 100 μL). The mice were euthanized at 2 or 7 h post infection. PLF sample was performed by washing the peritoneal cavity twice with PBS, yielding a total volume of 4 mL per mouse. PLF samples were centrifuged at 800 *g* for 5 min to pellet cells, followed by centrifugation of the supernatant at 6,000 *g* for 10 min to remove bacteria. The samples were filtered through 0.22 µm centrifuge tube filters, snap-frozen in liquid nitrogen, and stored at −80°C until further analysis.

Metabolites in the PLF were identified and quantified using high-resolution liquid chromatography-mass spectrometry (LC-MS) at the Genepioneer Metabolomics Research Facility (Genepioneer, China). Chromatography separation and mass spectrometry detection were carried out on an Orbitrap Exploris 120 instrument (Thermo Fisher Scientific, USA) following previously described methodologies (60, 61). Metabolite identification was tentatively assigned based on monoisotopic mass and MS/MS fragmentation patterns. Theoretical accurate masses were computed using ChemDraw (v12.0, CambridgeSoft), and instrument control, data acquisition, and qualitative analysis were performed with Xcalibur software (v2.1, Thermo Fisher Scientific).

Raw data were processed by matching experimental spectra against reference libraries from HMDB, KEGG COMPOUND, and KEGG PATHWAY (https://www.kegg.jp/kegg/pathway.html). Raw data from two independent metabolomic profiling experiments, including mock, SS2-2 hpi, SS2-7 hpi, Δ*sly*-2 hpi, Δ*sly*-7 hpi, and rSLY-2 hpi groups, were merged and processed together for database retrieval (Table S1a and b). Metabolites with identification confidence levels ≥ Level 3.1 were retained, and a total of 850 high-confidence metabolites were obtained from the intersection across four groups (Mock vs SS2-2 hpi, Mock vs SS2-7 hpi, Mock vs Δ*sly*-2 hpi, and Mock vs Δ*sly*-7 hpi) for subsequent analysis (Table S1c through g). Based on the 850 high-confidence metabolites, unsupervised metabolite clustering was conducted as previously reported (62). Z-score was calculated from the original scale data, and partitioning around medoids (PAM) clustering was implemented using the R package cluster (v4.5.0) with the parameter (*k* = 7).

### Targeted metabolomics

Five- to six-week-old female BALB/c mice were injected with PBS (100 μL), SS2 (1 × 10^8^ CFU in 100 μL), Δ*sly* (1 × 10^8^ CFU in 100 μL), or rSLY (0.5 mg/kg in 100 μL). Animals were euthanized 7 h post infection. The peritoneal cavity was washed twice with PBS, and a total of 4 mL of PLF was collected. PLF samples were centrifuged at 800 *g* for 5 min to isolate the cells, followed by centrifugation at 6,000 *g* for 10 min to isolate the bacteria. The samples were filtered through 0.22 µm centrifuge tube filters, snap-frozen in liquid nitrogen, and stored at −80°C until further analysis.

Targeted metabolomic profiling of oxidized lipids in the PLF samples was conducted using high-performance Research Facility (Bioyigene, China). A total of 141 oxidized lipids were quantitatively analyzed with high sensitivity and broad coverage. Briefly, 200 μL of methanol/acetonitrile was added to each sample, which was then centrifuged at 12,000 *g* for 10 min. the supernatant was dried under a nitrogen stream, and the residue was reconstituted in 100 μL of water/methanol (1:1, vol/vol) with thorough vortexing. LC-MS/MS analysis was performed using QTRAP 6500+ system (SCIEX) equipped with an electrospray ionization source in both positive and negative modes. Chromatographic separation was achieved on an HSS T3 C18 column (2.1 mm × 100 mm, 1.8 μm, Waters). Raw data were processed using Analyst software (v1.6.3) for peak integration, calibration, and metabolite quantification.

### Cytokine expression analysis

To evaluate the effect of SLY and AA on pro-inflammatory cytokine expression *in vivo*, 5- to 6-week-old female BALB/c mice were injected intraperitoneally with rSLY (7.5 mg/kg), AA (12.5 mg/kg), or an equal volume of PBS (mock control). At 7 h post-injection, mice were euthanized, and the peritoneal cavity was lavaged twice with 4 mL PBS. PLF was collected and centrifuged at 800 *g* for 5 min to isolate cells. Total RNA was extracted from the cell pellets using Trizol reagent (Vazyme, China). Two hundred microliters of chloroform was added per 1 mL Trizol, and samples were vortexed vigorously. The supernatant was added to an equal volume of 70% ethanol and mixed. RNA purification was performed with a E.Z.N.A. Total RNA kit I (Omega, USA). RNA from each sample was converted to cDNA using the HiScript II RT SuperMix for qPCR (Vazyme, China). cDNA was diluted 10-fold and subjected to real-time PCR amplification using AceQ qPCR SYBR Green Master Mix (Vazyme, China) with specific primers.

β-Actin-F: TTCTTTGCAGCTCCTTCGT

β-Actin-R: ATGGAGGGGAATACAGCCC

IL-1β-F: TCGTGGCTGTCGGACCCATAT

IL-1β-R: GCCCAAGGCCACAGGTATTT

IL-18-F: GGACACTTTCTTGCTTGCCA

IL-18-R: ATCATGCAGCCTCGGGTATT

TNFα-F: CCAAATGGCCTCCCTCTCAT

TNFα-R: GGTGGTTTGCTACGACGTGG

### Statistics

All statistical analyses were performed with GraphPad Prism 9.5.0, and *T* test, one-way ANOVA, and two-way ANOVA were used to analyze the statistical significance. *P* < 0.05 were considered statistically significant.

## Data Availability

The STAMPR raw data that support the findings of this study are publicly available from the National Center for Biotechnology under accession code BioProject ID PRJNA1371331.

## SUPPLEMENTAL MATERIAL

Supplemental material iai.00164-26-s0001.docx Fig. S1 to S7; Table S2.

Table S1 iai.00164-26-s0002.xlsx Metabolites identified by untargeted metabolomics.

ASM does not own the copyrights to Supplemental Material that may be linked to, or accessed through, an article. The authors have granted ASM a non-exclusive, world-wide license to publish the Supplemental Material files. Please contact the corresponding author directly for reuse.
