# Supplementary material for: The cholesterol-dependent cytolysin promotes Streptococcus systemic spread and induces arachidonic acid accumulation-mediated lethality in a murine intraperitoneal infection model
Source: Infect Immun. 2026 Jun 22;94(7):e00164-26. doi: 10.1128/iai.00164-26 (PMC13367039; doi:10.1128/iai.00164-26)
Supplement: Supplemental material — Fig. S1 to S7; Table S2. [file iai.00164-26-s0001.docx]

**The cholesterol-dependent cytolysin promotes *Streptococcus* systemic spread and induces arachidonic acid accumulation mediated lethality in a murine intraperitoneal infection model**

Linya Xia^1#^, Xingyu Tian^1#^, Chen Yuan^1^, Zhaoxuan Zhu^1^, Fei Pan^1^, Hongjie Fan^1,4^, Karthik Hullahalli^2,3*^, Matthew K. Waldor^2^, Zhe Ma^1,4^**^*^**

1. Ministry of Agriculture Key Laboratory of Animal Bacteriology, the International Joint Laboratory of Animal Health and Food Safety, and College of Veterinary Medicine, Nanjing Agricultural University, Nanjing, Jiangsu 210095, China

2. Howard Hughes Medical Institute, Brigham and Women’s Hospital Division of Infectious Diseases, and Department of Microbiology, Harvard Medical School, Boston, MA 02115, USA

3. Department of Microbiology and Immunology, Stritch School of Medicine, Loyola University Chicago, Maywood, IL 60153, USA

4. Jiangsu Co-innovation Center for Prevention and Control of Important Animal Infectious Diseases and Zoonoses, Yangzhou, Jiangsu, 225009, China

* To whom correspondence may be addressed, E-mail: [mazhe@njau.edu.cn](mailto:mazhe@njau.edu.cn); khullahalli@luc.edu

^#^ These authors contribute equally to this work.

**Contents:**

Fig S1-S7

Tables S1-S2


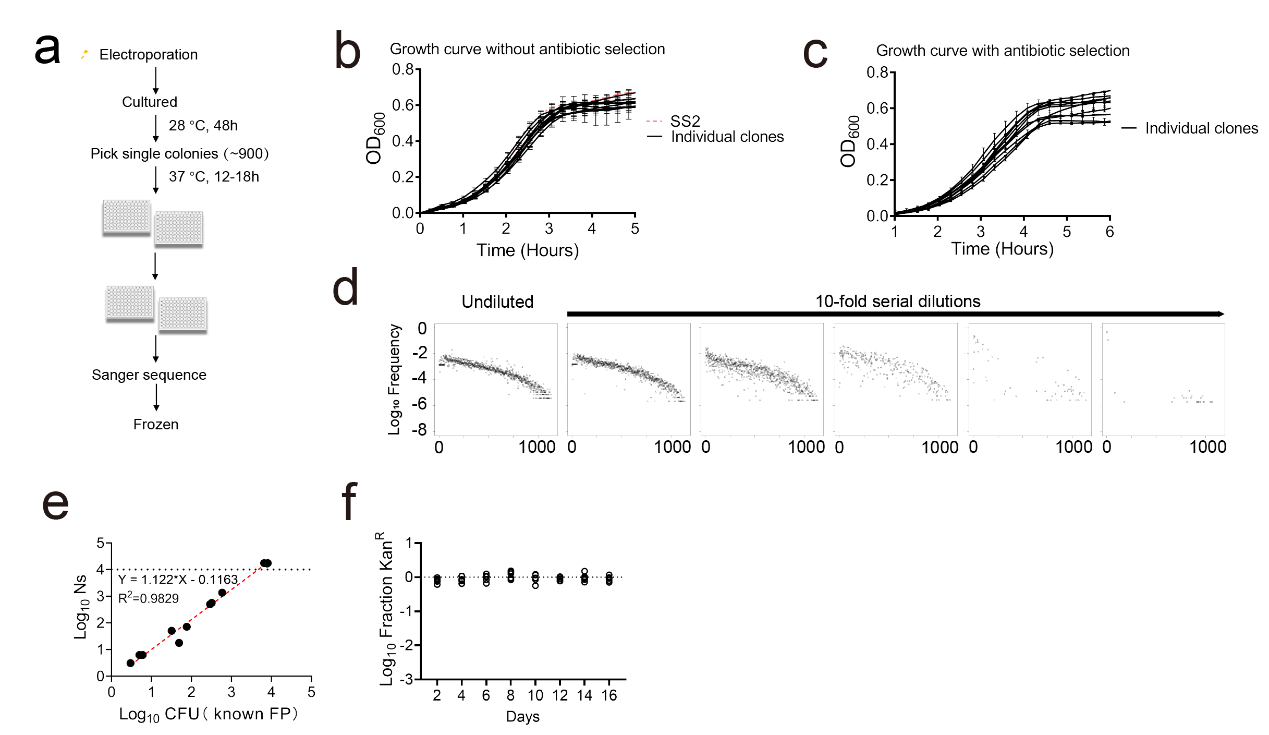


**S1 Fig.** **SS2 STAMP library construction and evaluation.**

**(a)** The schematic of the procedure of SS2 barcoded library construction. Individual barcoded bacteria were inoculated in single well of 96-well plates. ~900 individual colonies were pooled and frozen. **(b, c)** The growth curves of randomly picked 10 individual barcoded clones and the WT in THB medium without Kan (b) or with Kan (c). The barcode insertion did not cause growth defect in SS2. **(d, e)** Sequencing of the barcodes at various known bottleneck sizes demonstrated that Ns accurately reflect founding population (FP) values up to 10^4^. **(f)** The barcoded library was serially passaged in THB medium without Kan. Every two days, the colonies containing barcodes were counted as a fraction of the kanamycin-resistant cells. All colonies retained the antibiotic resistance marker linked to barcode, indicating that the barcode is stable for at least 16 days without Kan.


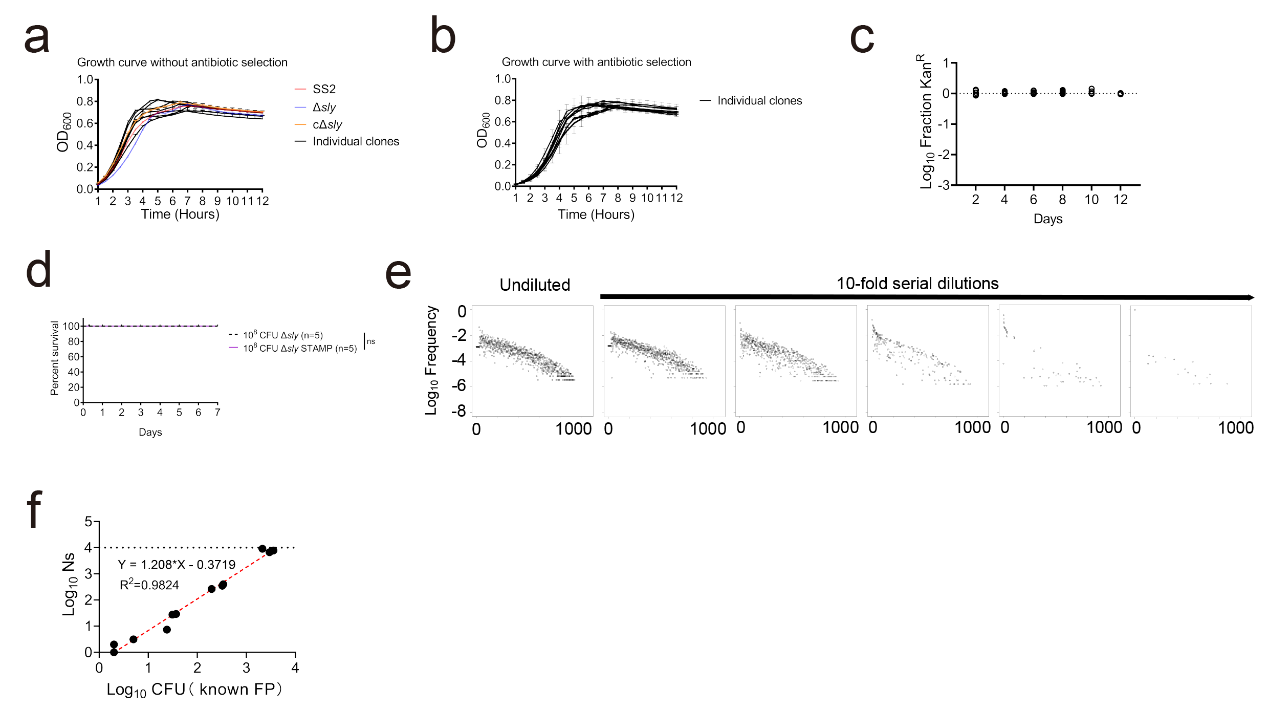


**S2 Fig. Δ*sly* STAMP library construction and evaluation.**

**(a)** The growth curves of 7 randomly individual barcoded Δ*sly* clones, the WT, Δ*sly* and cΔ*sly* in THB medium without Kan. Deletion of *sly* gene and insertion of barcode tags do not confer any growth defects. **(b)** The growth curves of 10 randomly picked barcoded-Δ*sly* clones in THB medium with Kan. **(c)** The Δ*sly* barcoded library was serially passaged in THB medium without Kan. Every two days, the colonies containing barcodes were counted as a fraction of the kanamycin-resistant cells. All colonies retained the antibiotic resistance marker linked to barcode, indicating that the barcode is stable for at least 12 days without Kan. **(d)** Survival curves of mice challenged with 1×10^8^ CFU of Δ*sly* or Δ*sly*-STAMP. Mice received 1×10^8^ CFU were monitored for 7 days and both groups exhibited a 100% survival rate. Survival curves were analyzed using the Log-rank (Mantel-Cox) test. ns, not significant. **(e, f)** Sequencing the Δ*sly* barcodes at various known bottleneck sizes. Ns accurately reflect FP values up to 10^4^.


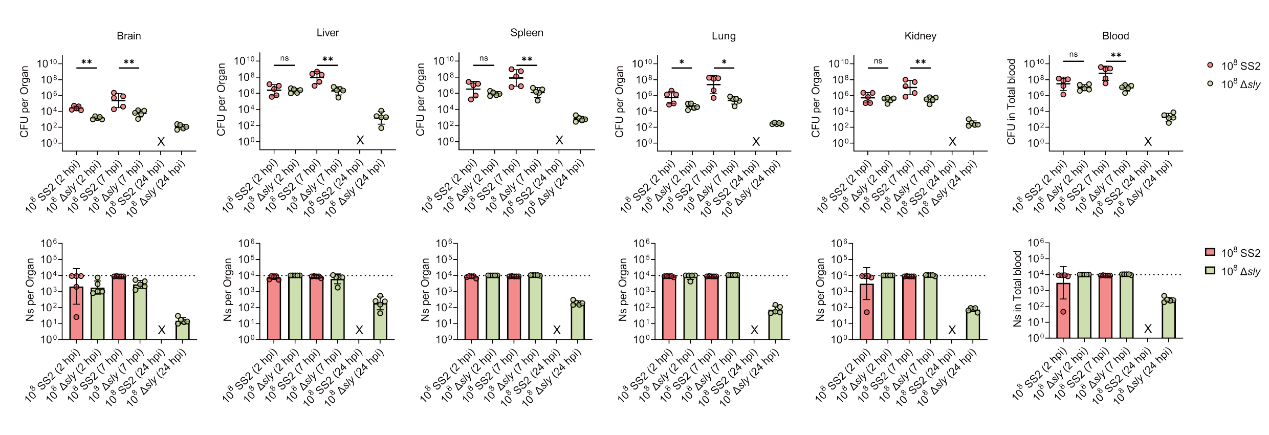


**S3 Fig. Infection dynamics analysis of barcoded library with 10^8^ CFU of libraries.**

Bacterial burden (Top) and Ns (Bottom) in organs and blood of mice were measured after challenging with 1×10^8^ CFU of WT-STAMP library at 2 hpi (n=5), 7 hpi (n=5), or 1×10^8^ CFU of Δ*sly*-STAMP library at 2 hpi (n=5), 7 hpi (n=5) and 24 hpi (n=5). Symbols are individual samples, and bars indicate geometric means with geometric SD. Dotted line at 10^4^ represents the resolution limit of the library. Statistical significance was calculated using nonparametric T test by Mann-Whitney U test. ns, not significant; ***p* < 0.01; **p* < 0.05.


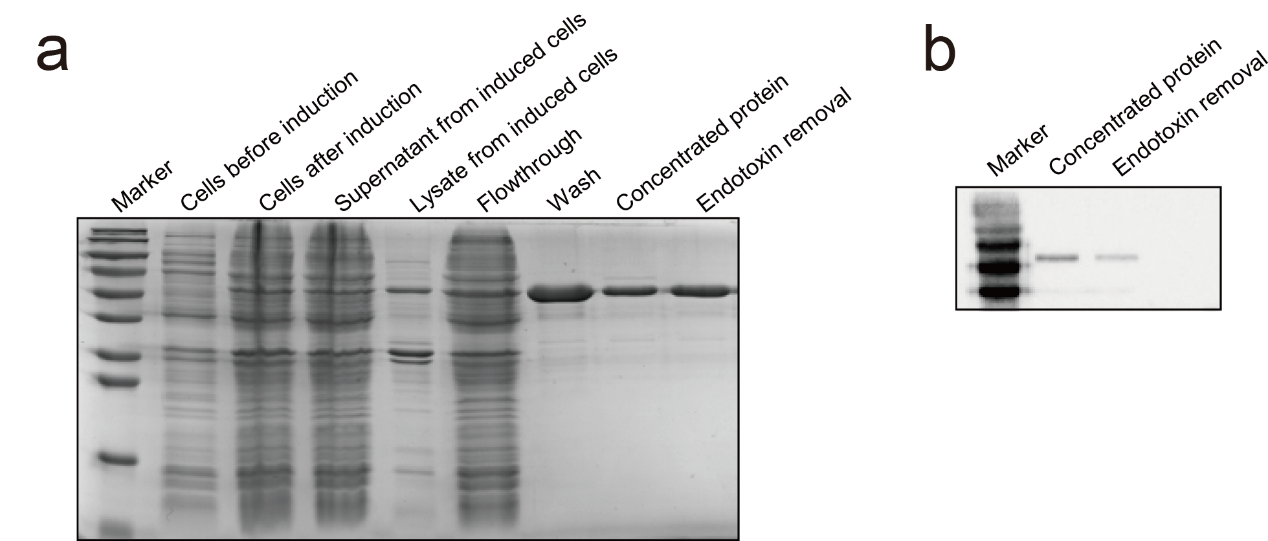


**S4 Fig. Expression and purification of recombinant SLY protein.**

**(a)** SDS-PAGE analysis of rSLY protein expressed in *E. coli* BL21(DE3) harboring pET28a-rSLY plasmid, showing induced expression, affinity chromatography–purified protein, and rSLY concentrated using a 10 kDa ultrafiltration device followed by endotoxin removal. (b) Western blot detection of concentrated rSLY protein before and after endotoxin removal.


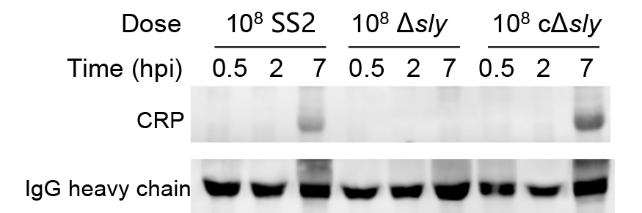


**S5 Fig. Western blot detection of CRP in the serum of mice after SS2 or Δ*sly* challenge.**

Immunoblot detection of CRP (25 kDa) and IgG heavy chain (as reference, 50 kDa) in mouse serum. Time course (0.5-7 hpi) of CRP expression following infection with SS2, Δ*sly* or cΔ*sly* strains at an inoculation dose of 1×10^8^ CFU. Serum samples were collected at 0.5, 2, and 7 hpi.


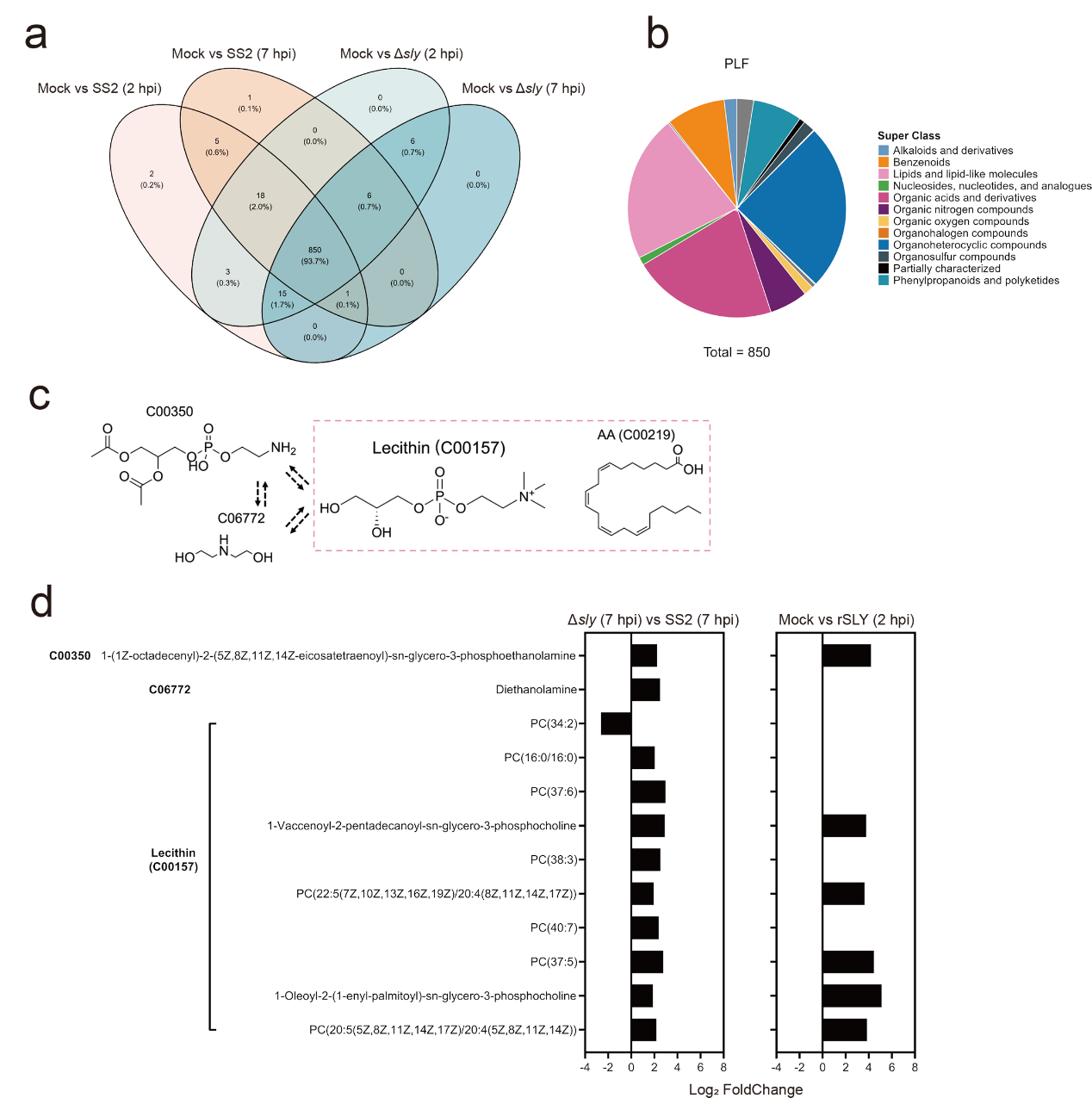


**S6 Fig. Suilysin induced lipid and lipid-like metabolites enrichment.**

**(a)** Components of a Venn diagram. Metabolites with identification confidence levels ≥ Level 3.1 were retained, and a total of 850 high-confidence metabolites were obtained from the intersection across four groups (Mock vs SS2-2 hpi, Mock vs SS2-7 hpi, Mock vs Δ*sly*-2 hpi, and Mock vs Δ*sly*-7 hpi) for subsequent analysis (Supplementary Table 1c-f). **(b)** Functional categories of 850 metabolites identified in the PLF of uninfected mice by untargeted metabolomic analysis. A complete list of metabolites in each category is provided in Supplementary Table 1g. **(c)** Metabolites identified by untargeted metabolomics mapped to the glycerophospholipid metabolism pathway. Lecithin (C00157) serves as the primary precursor of arachidonic acid (AA; C00219). **(d)** Differential metabolite abundance in panel (c) between Δ*sly* (7 hpi) vs SS2 (7 hpi) groups and between Mock vs rSLY (2 hpi) groups.


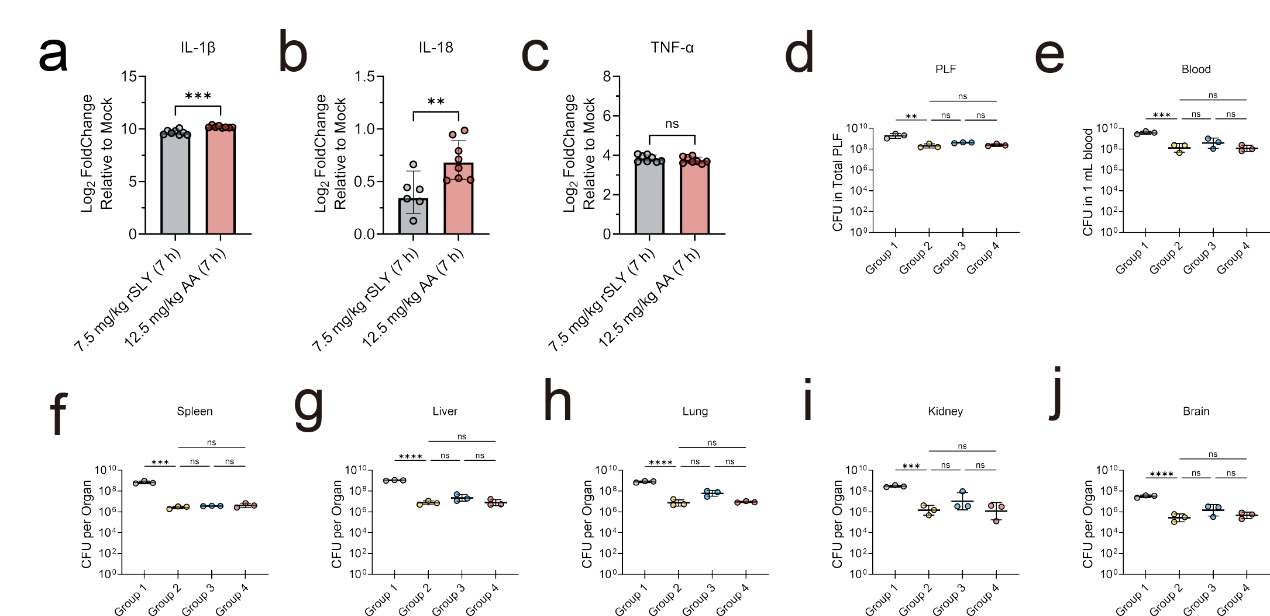


**S7 Fig. PLA_2_ inhibition mitigates AA-driven inflammatory pathology during SS2 infection.**

**(a-c)** qPCR analysis of pro-inflammatory cytokine mRNA levels (IL-1β, IL-18, and TNF-α) in PLF cells at 7 hpi. Mice were injected intraperitoneally with PBS (mock), AA (12.5 mg/kg), or rSLY (7.5 mg/kg). Expression was normalized to that of β-Actin. IL-1β, IL-18, and TNF-α gene expression relative to mock is presented as the log_2_ FoldChange ratio. Data are presented as geometric means with geometric SD of three independent experiments. Statistical significance was calculated using nonparametric T test by Welch’s T test. ns, not significant; ****p* < 0.001; ***p* < 0.01. **(d-j)** Bacterial CFUs in the PLF, blood and other organs from mice challenged with 1×10^8^ CFU of SS2 at 7 hpi. Mice were injected intraperitoneally with PBS (Group 1, n=3), 500 mg/kg Spc alone (Group 2, n=3), 500 mg/kg Spc and 10 mg/kg cPLA_2_ inhibitor (Group 3, n=3), or 500 mg/kg Spc and 10 mg/kg sPLA_2_ inhibitor (Group 4, n=3) at 2 hpi. Bars indicate geometric means with geometric SD. Statistical significance was calculated using one-way ANOVA by Tukey test. The ns indicates no significant difference, *****p* < 0.0001; ****p* < 0.001; ***p* < 0.01.

**S1 Table.** **Untargeted metabolomic analysis of** **metabolites identified in the PLF of mice.**

| **Score** | **0** | **1** | **2** | **3** |
| --- | --- | --- | --- | --- |
| Mesothelial Integrity | Single layer, continuous, no denudation | Focal denudation < 25% | Patchy denudation 25 - 75% | Extensive denudation > 75% or complete loss |
| Inflammatory Cell Infiltration | None or minimal | Scattered isolated cells | Multifocal aggregates | Dense, diffuse, transmural involvement |
| Fibrosis/Collagen Deposition | SMC^+^ thickness < 50 μm | 50 - 150 μm | 150 - 300 μm | > 300 μm or prominent collagen/α-SMA^+^ fibroblast proliferation |
| Vascular Changes /Neovascularization | Density comparable to control | Vascular density increased < 1.5-fold | Vascular density increased 1.5 - 2-fold | Vascular density increased > 2-fold or marked dilation/hyperplasia |
| Fibrin Deposition | None | Thin layer on surface | Sheet-like deposition | Thick layer/covering organ spaces |
| Edema | None | Mild interstitial vacuolation | Moderate vacuolation with collagen separation | Marked vacuolation, tissue dissociation |
| Necrosis/ Hemorrhage | Absent | Focal | Multifocal | Diffuse or necrosis with hemorrhagic patches |

**S2 Table.** **The parietal peritoneum histology scoring system.**
